# A New Image Classification Approach via Improved MobileNet Models with Local Receptive Field Expansion in Shallow Layers

**DOI:** 10.1155/2020/8817849

**Published:** 2020-08-01

**Authors:** Wei Wang, Yiyang Hu, Ting Zou, Hongmei Liu, Jin Wang, Xin Wang

**Affiliations:** ^1^College of Computer and Communication Engineering, Changsha University of Science and Technology, Changsha 410114, China; ^2^Yiyang Branch, China Telecom Co., Ltd., Yiyang 413000, China; ^3^Hunan Railway Professional Technology College, Zhuzhou 410116, China; ^4^School of Information Science and Engineering, Fujian University of Technology, Fujian 350118, China

## Abstract

Because deep neural networks (DNNs) are both memory-intensive and computation-intensive, they are difficult to apply to embedded systems with limited hardware resources. Therefore, DNN models need to be compressed and accelerated. By applying depthwise separable convolutions, MobileNet can decrease the number of parameters and computational complexity with less loss of classification precision. Based on MobileNet, 3 improved MobileNet models with local receptive field expansion in shallow layers, also called Dilated-MobileNet (Dilated Convolution MobileNet) models, are proposed, in which dilated convolutions are introduced into a specific convolutional layer of the MobileNet model. Without increasing the number of parameters, dilated convolutions are used to increase the receptive field of the convolution filters to obtain better classification accuracy. The experiments were performed on the Caltech-101, Caltech-256, and Tubingen animals with attribute datasets, respectively. The results show that Dilated-MobileNets can obtain up to 2% higher classification accuracy than MobileNet.

## 1. Introduction

Computer image classification is one of the research hotspots in the field of computer vision. It can replace human visual interpretation to some extent by analyzing the image and classifying it into one of several categories. Image classification research mainly focuses on image feature extraction and classification algorithm. The features are very critical to the image classification, but traditional image features such as SIFT [1], HOG [2], and NSCT [3] are usually manually designed. So, the traditional methods are difficult to meet the requirements of the designer. On the contrary, convolutional neural network (CNN) can automatically extract features by using the prior knowledge of known image samples. It can avoid the complex feature extraction process in traditional image classification methods, and the extracted features have strong expression ability and high classification efficiency.

Deep learning technologies [4, 5] have been increasingly applied in image classification [6], target tracking [7], object detection [8], image segmentation [9, 10], and so on, all of which have achieved good results. Russakovsky et al. [11] used AlexNet of approximately 60 million parameters with 5 convolutional layers and 3 fully connected layers to win the 2012 champion of ImageNet Large-scale Visual Recognition Challenge. Then, in order to achieve higher classification accuracy, the deep neural network (DNN) structures have become deeper and more complex. For example, VGG [12] deepened the network to 19 layers, GoogleNet [13] used inception as the basic structure (the network reaches 22 layers), and ResNet [14] introduced residual network structure to solve the gradient vanishing problem. However, the complex DNNs have a large number of parameters and a large amount of computation, which requires a lot of memory access and CPU/GPU resources. Some real-time applications and low-memory portable devices still cannot fully meet the resource requirements of the DNN models.

To solve the above problems, more and more researches have focused on lightweight networks, which have fewer parameters and less computation while maintaining high accuracy. When analyzing the number of network parameters, Denil et al. [15] found that the parameters in the deep network have a lot of redundancy. In the process of processing, these parameters were useless to improve the classification accuracy but affected the processing efficiency. Hinton et al. [16] significantly improved the compressed model by distilling the models' ensemble knowledge. The classification accuracy of this simple network was almost as same as that of complex network. In terms of network compression, Iandola et al. [17] proposed a small CNN structure called SqueezeNet in 2016, which greatly reduced the number of network parameters. By using depthwise Separable Filters, Howard et al. [18] designed a streamlined architecture called MobileNet, based on depthwise convolution filters and pointwise convolution filters. MobileNet used two global hyperparameters to keep a balance between efficiency and accuracy. As an extremely computation-efficient CNN architecture, ShuffleNet [19] adopted two new operations, pointwise group convolution and channel shuffle. This network can be applied to mobile devices with very limited computing power.

Although the parameters or computation of lightweight network is reduced, the accuracy of classification also decreases correspondingly. Therefore, by introducing the dilated convolution filter into MobileNet, a Dilated-MobileNet approach is proposed based on local receptive field expansion. Without increasing the parameters, the dilated convolution filter can make the network obtain larger local receptive field and improve the classification accuracy.

## 2. Fundamental Frameworks

### 2.1. CNN Structure

**Figure 1:**
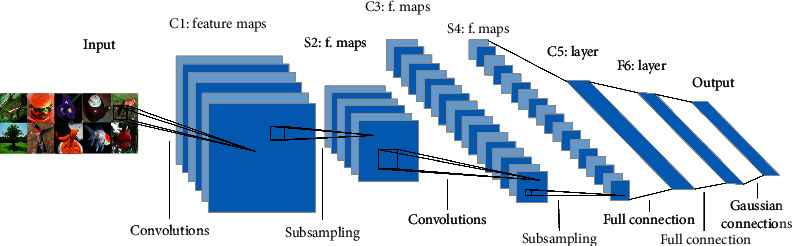
The basic structure of convolution neural network [21].

Convolutional neural network usually consists of convolutional layer, pooling layer, and full connection layer [20], as shown in Figure 1. First, the features are extracted by one or more convolution layers and pooling layers. Then, all the feature maps from the last convolution layer are transformed into one-dimensional vectors for full connection. Finally, the output layer classifies the input images. The network adjusts the weight parameters by back propagation and minimizing the square difference between the classification results and the expected outputs. The neurons in each layer are arranged in three dimensions: width, height, and depth, in which width and height are the size of neurons, and depth refers to the channels number of the input picture or the number of input feature maps.

The convolutional layer, which contains several convolution filters, extracts different features from the image by convolution operation. The convolution filters of the current layer convolute the input feature maps to extract local features and get the output feature maps. Then, the nonlinear feature maps can be obtained by using activation function.

The pooling layer, also known as the subsampling layer, is behind the convolutional layer. It performs downsampling operation, using a specific value as output in a certain subregion. By removing the unimportant sample points from the feature map, the size of input feature map of the subsequent layer is reduced, and the computational complexity is also diminished. At the same time, the adaptability of the network to the changes of image translation and rotation is increased. The most common pooling operations are maximum pooling and average pooling.

The structure based on convolutional layer and pooling layer can improve the robustness of the network model. The convolutional neural network can get deeper through multilayer convolutions. With the number of layers increasing, the features achieved through learning become more global. The global feature map learned at last is transformed into a vector to connect the full connection layer. Most of the parameters in the network model are at the full connection layer.

### 2.2. MobileNet Structure

**Figure 2:**
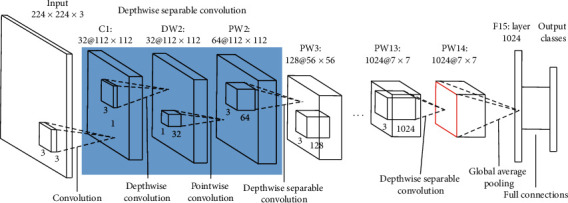
Architecture of MobileNet.

MobileNet, as shown in Figure 2, has smaller structure, less computation, and higher precision, which can be used for mobile terminals and embedded devices. Based on depthwise separable convolutions, MobileNets use two global hyperparameters to keep a balance between efficiency and accuracy.

**Figure 3:**
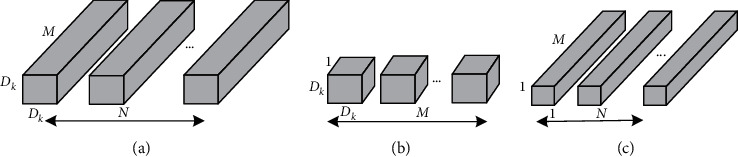
(a) Standard convolution filters, (b) depthwise convolution filters, and (c) pointwise convolution filters.

The core idea of MobileNet is the decomposition of convolution kernels. By using depthwise separable convolution, the standard convolution can be decomposed into a depthwise convolution and a pointwise convolution with 1 × 1 convolution kernel, as shown in Figure 3. The depthwise convolution filters perform convolution to each channel, and the 1 × 1 convolution is used to combine the outputs of the depthwise convolution layers. In this way, *N* standard convolution kernels (Figure 3(a)) can be replaced by *M* depthwise convolution kernels (Figure 3(b)) and *N* pointwise convolution kernels (Figure 3(c)). A standard convolutional filter combines the inputs into a new set of outputs, while the depthwise separable convolution divides the inputs into two layers, one for filtering and the other for merging.

## 3. Dilated-MobileNet (Dilated Convolution MobileNet) Structure

MobileNet (Figure 2) mostly uses 3 × 3 convolution filters. Although this network can reduce the computation cost, the local receptive fields of small convolution filter are too small to capture better features in the case of higher resolution of the feature maps. However, using large convolution filters will increase the number of parameters and the computation load. Therefore, in some first shallow convolutional layers, we use the dilated convolution with the expansion rate of 2 instead of the standard convolution. We call this network Dilated Convolution MobileNet (Dilated-MobileNet).

### 3.1. Dilated Convolution

Dilated convolution filter [22], which was first applied in image segmentation, is a kind of convolution filter which inserts 0 values between the adjacent nonzero values in feature maps. Image segmentation needs the same size image as the original input image, but the pooling layer in traditional DNN will reduce the spatial resolution of the feature map. In order to generate an effective dense feature map and obtain the same size of receptive field, Chen et al. [10] removed the maximum pooling layer in last layers of the full CNN and added dilated convolution. This method not only avoids the reduction of the spatial resolution of the feature map in the pooling layer but also increases receptive field as same as the pooling layer does.

**Figure 4:**
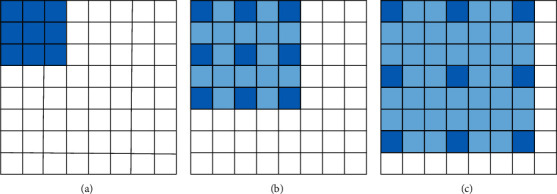
Schematic diagram of dilated convolution kernel.

The dilated convolution filter expands the receptive field by inserting 0 values between the nonzero values, as shown in Figure 4. Figure 4(a) represents the receptive field of a 3 × 3 convolution filter. Figure 4(b) indicates the receptive field, while the 3 × 3 convolution kernel changed to 5 × 5 when the expansion rate is 2. Figure 4(c) shows the receptive field, while the 3 × 3 convolution kernel changed to 7 × 7 when the expansion rate was 3. Therefore, the dilated convolution can expand the receptive field of convolution filter without increasing the parameters of convolution filter.

### 3.2. Dilated-MobileNet

Receptive field refers to the size of each element in the feature map of every layer's output mapped on the input image, so the layer will have larger receptive field when closer to the bottom of the network, and its receptive field is approximately equal to the global receptive field. In our research, expanding local receptive field is to improve the classification accuracy of MobileNet, so the layers which need increasing receptive field are near the input of the MobileNet. According to the location of the dilated convolution filter, we propose 3 new network models named D1-MobileNet, D2-MobileNet, and D3-MobileNet.

#### 3.2.1. Dilated1-MobileNet

**Figure 5:**
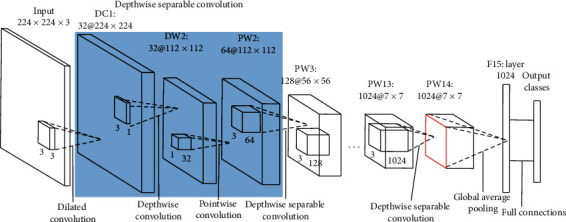
Architecture of Dilated1-MobileNet.

D1-MobileNet sets convolutional stride as 1 in the first layer and replaces the standard convolution filters with dilated convolution filters with an expansion rate of 2. At the same time, in order to restrain the increase of calculation cost, the stride of the 2nd depthwise separable convolution is set as 2, and the other layers remain unchanged. Compared with MobileNet, the first convolutional layer with stride 1, the size of the output feature map of the first convolutional layer changes from 112 × 112 to 224 × 224, as shown in Figure 5.

#### 3.2.2. Dilated2-MobileNet

**Figure 6:**
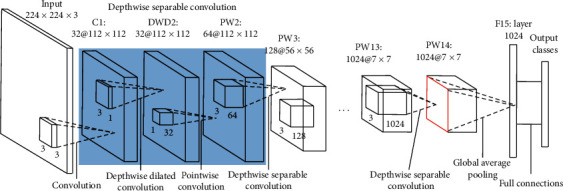
Architecture of Dilated2-MobileNet.

In DWD2 (depthwise separable) layer, the depthwise convolution filters is expanded by dilated convolution filters with an expansion rate of 2, while the other layers remain unchanged. This approach does not increase the amount of computation and parameters nor does it change the size of the output feature map of any layer, as shown in Figure 6.

#### 3.2.3. Dilated3-MobileNet

**Figure 7:**
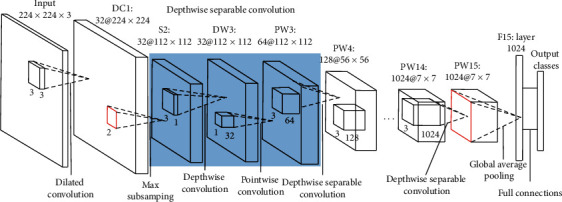
Architecture of Dilated3-MobileNet.

D3-MobileNet sets the convolutional stride in first convolutional layer as 1 and replaces the standard convolution filters with dilated convolution filters by using an expansion rate of 2. After the convolution operation in the first convolution layer, it is normalized through batch normalization layer [23]. Then, a maximum pooling layer with a stride of 2 is behind the batch normalization layer, and the other layers are unchanged, as shown in Figure 7.

In terms of receptive field expansion, there are also different ways of expansion. For example, Sun W combined dilated convolution and depthwise separable convolution to form standard blocks for network construction [21]. Their approach is to add a dilated convolution layer before each depthwise separable convolution. Unlike their approach, in the Dilated1-MobileNet, we use dilated convolution instead of the standard convolution in the first layer of MobileNet, without adding dilated convolution in front of all subsequent depthwise separable convolution blocks because that would increase the number of parameters. The difference in Dilated2-MobileNet is greater because we extend the receptive field in depthwise convolution layer rather than adding a dilated convolution layer in front of the depthwise separable convolution layer. Similarly, Dilated3-MobileNet replaces standard convolution with a dilated convolution at the first level and add a pooling layer after it, rather than adding a dilated convolution in front of all depthwise separable convolution blocks.

### 3.3. Computation Analysis

In the standard convolutional layer, assuming the height, width, and input channel number of the input feature maps *I* are *h*, *w*, and *m*, the convolution filter *K* is *s* × *s*, the output channel number is *n*, and the output feature maps *O*=*K* × *I* can be obtained by the convolution of *I* and *K* with no padding zeros and stride 1, as shown in the following formula:

(1) $$\begin{array}{c} O\left(y,x,j\right)=\sum_{i=1}^{m}\sum_{u,v=1}^{s}K\left(u,v,i,j\right)I\left(y+u-1,x+v-1,i\right), \end{array}$$

where *O*(*y*, *x*, *j*) represents the value of point (*y*, *x*) in *j*th output feature map, *K*(*u*, *v*, *i*, *j*) represents the value of point (*u*, *v*) on channel *i* in *j*th convolution filter, and *I*(*y*, *x*, *i*) represents the value of point (*y*, *x*) on *i*th input feature map. From Formula (1), it is known that an output value needs *s* × *s* × *m* times multiplication, so the total amount of calculations is *s* × *s* × *m* × (*h* − *s*+1) × (*w* − *s*+1) × *n* and the number of parameters is *s* × *s* × *m* × *n*.

When Dilated-MobileNet introduces the dilated convolution in the standard convolution layer, with feature map I, the dilated convolution is performed with no padding zeros by using convolution kernel *K* of the same size and expansion rate of 2. So, we can get the output feature map *O*_*d*_ by the following formula:

(2) $$\begin{array}{c} O_{d}\left(y,x,j\right)=\sum_{i=1}^{m}\sum_{u,v=1}^{s}K\left(u,v,i,j\right)I\left(y+u+\left(u-1\right)\left(r-1\right)-1,x+v+\left(v-1\right)\left(r-1\right)-1,i\right). \end{array}$$

So, the total computational amount of the dilated convolution layer is (*s* × *s* × *m*) × (*h* − *s* − (*s* − 1)(*r* − 1)+1) × (*w* − *s* − (*s* − 1)(*r* − 1)+1) × *n*, and the number of parameters is *s* × *s* × *m* × *n*. With no padding zeros, the computation of dilated convolution with expansion rate *r* > 1 is less than that of standard convolution, and the number of parameters is the same, but the receptive field of dilated convolution is larger than that of standard convolution. Under the convolution operation with padding zeros, the map size of the dilated convolution is the same as that of the standard convolution, both of which are *h* × *w* × *n*, and the computation and the number of parameters are the same too.

When introducing dilated convolution filters to the depthwise convolution, the above feature maps *I* is firstly convoluted with the depthwise convolution filter K, and the output feature graph *O*_*dc*_ is obtained through the following formula:

(3) $$\begin{array}{c} O_{dc}\left(y,x,j\right)=\sum_{u,v=1}^{s}K\left(u,v,j\right)I\left(y+u+\left(u-1\right)\left(r-1\right)-1,x+v+\left(v-1\right)\left(r-1\right)-1,j\right), \end{array}$$

where *O*_*dc*_(*y*, *x*, *j*) represents the value of point (*y*, *x*) in *j*th feature map. Since the depthwise convolution filter has only one channel, *K*(*u*, *v*, *j*) represents the value of point (*u*, *v*) on *j*th convolution filter and *I*(*y*, *x*, *j*) represents the value of point (*y*, *x*) on *j*th input channel.

The total computation of the depthwise separable convolution is (*s* × *s* × *n*) × (*h* − *s* − (*s* − 1)(*r* − 1)+1) × (*w* − *s* − (*s* − 1)(*r* − 1)+1) × *m*, and the total number of parameters is *s* × *s* × *m*+*m* × *n*. It can be seen that the parameter of the depthwise separable convolution are reduced compared with the standard convolution:

(4) $$\begin{array}{c} \frac{s\times s\times m+m\times n}{s\times s\times m\times n}=\frac{1}{n}+\frac{1}{s^{2}}. \end{array}$$

The ratio of computation is

(5) $$\begin{array}{c} \frac{\left(s\times s+n\right)\times \left(h-s-\left(s-1\right)\left(r-1\right)+1\right)\times \left(w-s-\left(s-1\right)\left(r-1\right)+1\right)\times m}{s\times s\times m\times n\times \left(h-s+1\right)\times \left(w-s+1\right)}=\frac{1}{n}+\frac{1}{s^{2}}. \end{array}$$

Similarly, when carrying out the depthwise convolution with padding zeros, the reduction ratio of parameters is

(6) $$\begin{array}{c} \frac{\left(s\times s+n\right)\times m\times h\times w}{s\times s\times m\times n\times h\times w}=\frac{1}{n}+\frac{1}{s^{2}}. \end{array}$$

From the above analysis, it can be seen that the receptive field of the deep convolution kernel with expansion rate *r* and convolution kernel size *s* × *s* is equivalent to that of the convolution kernel (*r* × *s* − *r*+1) × (*w* × *s* − *r*+1), thus can expand the receptive field without increasing the number of parameters and calculation amount.

### 3.4. Receptive Field

In many tasks, especially intensive prediction tasks such as semantic image segmentation and optical flow estimation, it is necessary to predict each pixel's value of the input image, and each output pixel's value needs a large receptive field to retain important information. Local receptive field refers to the size of the region in the input feature map of the upper layer, and the region is mapped by the pixel in the output feature map. In this paper, dilated convolution is used to enlarge the local receptive field of a certain layer to capture better features and further influence the receptive field size of the convoluted layer behind. The size of receptive field of each layer is shown in in the following formula:

(7) $$\begin{array}{c} r_{k}=\left\{\begin{array}{cc} f_{k}, & k=1,\\ r_{k-1}+\left(\left(f_{k}-1\right)\times \prod_{i=1}^{k-1}s_{i}\right), & k>1, \end{array}\right. \end{array}$$

where *r*_*k*_ denotes the receptive field size of the *k*th layer, *f*_*k*_ denotes the size of filter, and *s*_*i*_ denotes the stride of the *i*th layer. The receptive field of the first layer equals to the size of the filter. By using Formula (7), we can get the receptive field size of each layer of MobileNet and Dilated-MobileNet, as shown in Table 1.

The “ds” in Table 1 shows the depthwise separable convolution, and the pointwise convolution has the same receptive field as the depthwise convolution in depthwise separable convolution, so the receptive field is given uniformly. The receptive field sizes of the first convolution layers in D1-MobileNet and Dilated3-MobileNet show that the receptive field of the 3 × 3 convolution kernel changed to 5 × 5 when the expansion rate is 2. In summary, dilated convolution is able to enlarge the size of local receptive field. Moreover, Dilated1-MobileNet and Dilated2-MobileNet also slightly increase the receptive field size of the underlying layers. It can be seen from Table 1 that, for Dilated-MobileNet networks, although the expansion ratio of the receptive fields of the latter convolution layers becomes smaller, their receptive fields of the first few layers are larger than those of MobileNet. In this way, it is easier to extract more detailed information, which is conducive to the improvement of classification accuracy.

## 4. Experiments and Result Analysis

In the experiments, we compare the classification results of 6 networks: SqueezeNet [17], MobileNet [18], Dense1-MobileNet [24], Dense2-MobileNet [24], D1-MobileNet, D2-MobileNet, and D3-MobileNet on Caltech-101 [25] and Catech-256 [26] datasets and Tubingen Animals with Attributes [27].

**Figure 8:**
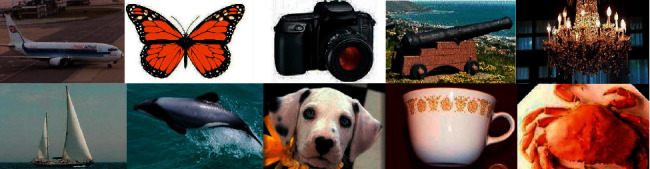
Picture instances in the Caltech-101 dataset.

The Caltech-101 dataset is an image object recognition dataset, which consists of a total of 9146 images, split between 101 different object classes and an additional background/clutter class. Each object class contains between 40 and 800 images on average. After labeling the pictures in the dataset, 1500 pictures are randomly selected as the test pictures and the rest as the training pictures. Some samples are shown in Figure 8.

**Figure 9:**
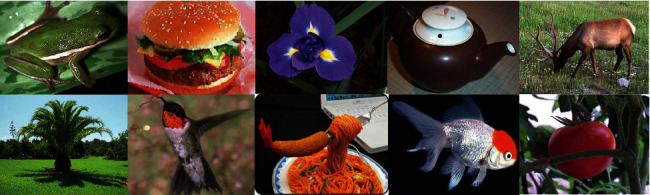
Picture instances in the Caltech-256 dataset.

The Caltech-256 dataset is based on the Caltech-101 dataset, adding image classes and the number of images in each class. The dataset contains 30607 images in 257 classes, including 256 object classes and one background class. Each class has at least 80 pictures and a maximum of 827 in background class.  Figure 9 shows the image examples in the Caltech-256 dataset. Each picture in the dataset is labeled and shuffled. 3060 pictures are randomly selected as test images, and the remaining pictures are used as training images.

**Figure 10:**
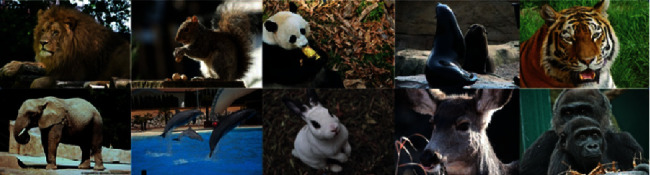
Picture instances in Tuebingen Animals (21) dataset.

We also verify our method on the Animals with Attributes (AwA) dataset, as shown in Figure 10. There are a total of 50 animal classes in the database with a total of 30475 pictures. In experiments, we select 21 animal categories, which are the largest classes and have almost the same number of pictures, as the experimental dataset. There are 22742 pictures in these 21 animal classes, and the number of pictures in each class is between 850 and 1600. After labeling the pictures in the dataset, 2000 pictures are randomly selected as the test pictures and the rest as the training pictures.

The experiments are under TensorFlow framework and the programming language is Python. The experimental server is equipped with an NVIDIA TITAN GPU. RMSprop optimization algorithm is used in the experiments. RMSprop is an adaptive learning rate method, which can adjust the learning rate. In the experiments, the initial learning rate is 0.1. Since the Xavier initialization method can determine the random initialization distribution range of parameters according to the number of inputs and outputs of each layer, we use it to initialize the weight coefficients. ReLU is used as the activation function in the experiments, and a total of 50,000 batches are trained, with 64 samples per batch.

In the following experiments, all the results are the averages of 10 times experiments, and the best classification accuracy rates are in bold in the tables.  Table 2 shows the classification accuracies of 7 network models on the Caltech-101 dataset.

As seen from Table 2, the accuracy rates of the 7 network models have reached a balance after 30000 iterations, and the accuracy rates of our 3 improved Dilated-MobileNets models are about 0.8%∼2% higher than those of the MobileNet model. Among of them, the classification accuracy rate of Dilated1-MobileNet model is improved by 0.87% and that of Dilated2-MobileNet model is improved by 1.13%. The Dilated3-MobileNet model has the best effect, the accuracy rate is increased by 2.13%, and the final classification accuracy rate is 78.73%.

Table 3 is a comparison of the classification accuracy rates of the 7 network models on the Caltech-256 dataset. As shown in Table 3, the accuracy rates of the 7 network models also have reached a balance after 30000 iterations, and the accuracy rates of our 3 improved models are improved by 0.5%∼1.5% than that of MobileNet model. Among of them, the accuracy rate of Dilated1-MobileNet model is improved by 1.35%, the accuracy rate of Dilated3-MobileNet model is improved by 0.64% and that of Dilated2-MobileNet model is the highest, which is improved by 1.42% and final reaches to 65.94%.

It can be seen from Table 4 that the accuracy rates of MobileNets and Dilated-MobileNet models have reached a balance after 30000 iterations, but the accuracy rate of SqueezeNet still increases and finally reaches a balance at the accuracy rate of 73.85% after 50000 iterations. As in the previous 2 experiments, the accuracy rates of MobileNet, Dense-MobileNets, and our 3 improved models are much higher than those of SqueezeNet. The accuracy rates of the 3 improved Dilated-MobileNet models are about 0.5%∼1.2% higher than those of MobileNet. Among them, the classification accuracy rate of Dilated1-MobileNet model is finally improved by 0.8%, the classification accuracy rate of Dilated2-MobileNet is finally improved by 0.4%, and the classification accuracy rate of Dilated3-MobileNet is the highest, reaching 92.8%.

In the above 3 kinds of experiments, the Dense1-MobileNet and Dense1-MobileNet based on dense connection also achieved good classification effect. The results of the experiments on caltech-256 dataset are slightly better than those of Dilated3-MobileNet and a little worse than those of Dilated1-MobileNet and Dilated2-MobileNet. The design idea of Dense-MobileNets is different from that of the Dilated-MobileNets, and the network structures are also different, so the two approaches can be used together in the practical application.3

## 5. Conclusions

The memory-intensive and highly computation-intensive properties of deep learning approaches restrict their applications in portable devices. At the same time, the compression and acceleration of network models will reduce the classification accuracy. So, this paper uses the dilated convolution in the lightweight neural network (MobileNet) to improve the classification accuracy without increasing the network parameters and proposes three Dilated-MobileNet models. The experimental results show that Dilated-MobileNets have better classification accuracies on Caltech-101, Catech-256, and AWA datasets.

In recent years, new lightweight networks, such as mobilenetv2 [29] and mobilenetv3 [28], have emerged. How to reduce the parameters and improve the classification effect is still one of the research hotspots. Meanwhile, some deep learning methods combined with traditional methods have achieved good results in target recognition and classification [30]. On the other hand, designing specific deep learning networks based on the characteristics of classification targets is a very effective classification approach [31, 32]. Therefore, how to give full use of the advantages of different methods is also worth further studying.

## Figures and Tables

**Table 1:** The receptive field size of each layer.

	MobileNet	Dilated1-MobileNet	Dilated2-MobileNet	Dilated3-MobileNet
Conv1	3	5	3	5
Pool	—	6	—	—
Conv2 ds	7	10	11	7
Conv3 ds	11	14	15	11
Conv4 ds	19	22	23	19
Conv5 ds	27	30	31	27
Conv6 ds	43	46	47	43
Conv7 ds	59	62	63	59
Conv8 ds	91	94	95	91
Conv9 ds	123	126	127	123
Conv10 ds	155	158	159	155
Conv11 ds	187	190	191	187
Conv12 ds	219	222	223	219
Conv13 ds	251	254	255	251
Conv14 ds	315	318	319	315

**Table 2:** Classification accuracy rates (%) on Caltech-101 dataset.

Number of iterations	30000	35000	40000	45000	50000
SqueezeNet	53.60	53.60	53.47	53.40	53.47
MobileNets	76.73	76.60	76.60	76.80	76.60
Dense1-MobileNet	76.60	76.53	76.47	76.40	76.47
Dense2-MobileNet	77.60	77.67	77.87	77.80	77.80
Dilated1-MobileNet	77.40	77.47	77.53	77.40	77.47
Dilated2-MobileNet	77.67	77.80	77.73	77.67	77.73
Dilated3-MobileNet	78.60	78.60	78.53	78.53	78.73

**Table 3:** Classification accuracy rates (%) on Caltech-256 dataset.

Number of iterations	30000	35000	40000	45000	50000
SqueezeNet	41.48	43.06	43.39	43.58	44.03
MobileNets	64.48	64.58	64.55	64.67	64.52
Dense1-MobileNet	64.61	64.53	64.45	64.44	64.47
Dense2-MobileNet	65.62	65.67	65.84	65.78	65.79
Dilated1-MobileNet	65.77	65.74	65.87	65.90	65.87
Dilated2-MobileNet	66.10	66.06	65.94	65.84	65.94
Dilated3-MobileNet	64.97	64.9	64.87	65.19	65.16

We also validate our method on the Animals with Attributes (AwA) dataset [28]. The classification accuracy rates are shown in Table 4.

**Table 4:** Classification accuracy rates (%) on AwA (21) dataset.

Number of iterations	30000	35000	40000	45000	50000
SqueezeNet	72.65	72.10	73.30	73.40	73.85
MobileNets	91.60	91.60	91.60	91.55	91.60
Dense1-MobileNet	90.65	90.60	90.60	90.60	90.65
Dense2-MobileNet	92.10	92.05	92.10	92.05	92.05
Dilated1-MobileNet	92.45	92.45	92.50	92.35	92.40
Dilated2-MobileNet	92.00	92.05	92.05	92.00	92.00
Dilated3-MobileNet	92.85	92.75	92.80	92.70	92.80

## Data Availability

All datasets in this article are public datasets and can be found on public websites.
